# Microbial community dynamics in rotational cropping: seasonality vs. crop-specific effects

**DOI:** 10.3389/fmicb.2025.1675394

**Published:** 2025-10-13

**Authors:** Debora Casagrande Pierantoni, Angela Conti, Laura Corte, Giacomo Tosti, Paolo Benincasa, Gianluigi Cardinali, Marcello Guiducci

**Affiliations:** ^1^CEMIN Excellence Research Centre, University of Perugia, Perugia, Italy; ^2^Department of Pharmaceutical Sciences, University of Perugia, Perugia, Italy; ^3^Department of Agricultural, Food and Environmental Sciences, University of Perugia, Perugia, Italy

**Keywords:** soil microbiome, crop rotation, seasonal variation, soil microbial diversity, microbial community dynamics

## Abstract

**Introduction:**

Soil microbial communities are central to soil health and plant productivity, yet their responses to crop rotation and seasonal changes remain incompletely understood. Understanding how crop identity and phenology shape these communities is essential for optimizing agricultural sustainability.

**Methods:**

This study examined how different crop species and their growth stages influence the diversity, composition, and functional characteristics of soil microbiota in a long-term crop rotation system. We integrated high-throughput DNA sequencing with soil chemical and spectroscopic analyses to assess microbial community dynamics across three key seasonal time points.

**Results:**

Our results indicate that while crop species and their growth stages can influence microbial community structure, these effects were generally modest and variable. In contrast, seasonal factors and soil physicochemical properties—particularly electrical conductivity—exerted stronger and more consistent effects on microbial beta diversity. Despite shifts in taxonomic composition, a core microbiome dominated by *Acidobacteriota* and *Bacillus* persisted across crops and seasons. Functional predictions revealed a seasonal peak in nitrification potential during warmer months, suggesting environmental rather than crop-driven control of this process.

**Discussion:**

These findings highlight the resilience of soil microbiomes under rotational systems and underscore the dominant role of seasonal and abiotic factors in shaping microbial community dynamics. A better understanding of these interactions can inform agricultural practices aimed at sustaining microbial functionality and promoting long-term soil health.

## 1 Introduction

Soil microbiota play a fundamental role in maintaining soil health, fertility, and ecosystem functioning (Churchland et al., 2013; Fierer, 2017). These diverse communities of bacteria and fungi are responsible for key processes such as nutrient cycling, organic matter decomposition, and the suppression of soil-borne diseases (Berendsen et al., 2012; Philippot et al., 2013). In agricultural systems, the structure and function of soil microbial communities are influenced by a complex interplay of factors, including soil properties, crop species, management practices, and seasonal dynamics (Hartmann et al., 2015; Schlatter et al., 2020; Zong et al., 2024).

Crop rotation is a widely adopted agricultural practice aimed at improving soil fertility, breaking pest and disease cycles, and enhancing crop productivity (Smith et al., 2007; Venter et al., 2016). However, the extent to which different crops and their growth stages affect soil microbial diversity and community composition remains a subject of ongoing research (García-Orenes et al., 2013; Lupatini et al., 2017) While it is well established that plant roots can shape the rhizosphere microbiome through the release of exudates and other mechanisms (Bais et al., 2006; Sasse et al., 2018; Seitz et al., 2024), the relative importance of crop identity vs. environmental factors such as soil type, pH, moisture, and seasonal changes is less clear (Lauber et al., 2009; Rousk et al., 2010).

Recent advances in high-throughput sequencing and molecular profiling have enabled more detailed investigations into how crop rotations and seasonal variation influence soil microbial communities (Caporaso et al., 2012; Mendes et al., 2015). These studies have revealed that, although plant species can exert some influence on the soil microbiota, their effects are often context-dependent and may be outweighed by abiotic factors and temporal variability (Thomson et al., 2010; Zhou et al., 2016). Understanding the drivers of soil microbial stability and change is crucial for developing sustainable agricultural practices that support long-term soil health and productivity (Schloter et al., 2018; Banerjee et al., 2019).

This study aims to elucidate how different crops and their seasonal growth stages affect the diversity, composition, and functional potential of soil microbiota within a long-term crop rotation system. By integrating high-resolution metabarcoding with soil chemical profiling, we seek to disentangle the relative contributions of crop species, vegetation stage, and environmental variables to soil microbial community dynamics. The findings will provide insights into the resilience and stability of the soil microbiome in response to agricultural management, with implications for optimizing crop rotations and promoting agroecosystem sustainability.

## 2 Materials and methods

### 2.1 Site description and sample collection

#### 2.1.1 Site description

This study utilized soil samples collected in 2019 from the Biosyst long-term experiment (LTE), conducted at the FIELDLAB of the University of Perugia (42°57′26.2″N, 12°22′21.2″E, 168 m a.s.l.). The samples were taken from the field managed under the conventional low-input system throughout the study (CON, EU reg. 2078/92), where the soil in the field was identified as Fluventic Haplustept, consisting of 26.3 ± 1.3% sand, 38.5 ± 1.2% silt, 35.2 ± 1.0% clay, with a pH of 8.04 ± 0.11 and 1.46 ± 0.05% soil organic matter (SOM).

Starting from October 2001, a 6-year crop rotation had been implemented with a fixed sequence of crops: (1) corn (C), (2) processing tomato (T), (3) durum wheat (DW), (4) field bean (FB), (5) muskmelon (M), and (6) common wheat (CW). To accommodate all the six crops in each year, the field was divided into six sectors, and six different crop rotation arrangements (R1–R6) were used, each starting with a different crop. For example, in the 2018/2019 season, after three full rotation cycles (i.e., 18 years), the crops in each sector were as follows: durum wheat in R1, soft wheat in R2, muskmelon in R3, processing tomato in R4, corn in R5, and field bean in R6. Soil sampling was performed using a 4 cm diameter core drilling tool to collect soil from the top 0–20 cm layer. In each sector (R1–6, i.e. for each culture), four sampling points were selected in the core area (excluding border furrows and plot edges), each sampling represents the biological replicates. At each sampling point, four cores were taken randomly, when the crop was not present, or in between two contiguous crop furrows, when crop furrows were visible. The four cores from each sampling point were mixed in a sterile plastic bag and stored at −80 °C for further analysis, improving sample homogeneity and minimizing spatial micro-variability. These pooled samples were not considered independent pseudo replicates but rather a single biological replicate. Each sector was treated independently, with no overlap in sampling points between sectors, ensuring statistical independence among replicates for a given crop. Sampling took place on three dates: March 27th, June 20th and September 6th, 2019. A schematic description of the experiment in the year 2018/2019, including crop growth stages, are reported in Supplementary Figure 1 and Table 1. A detailed description of the field management and condition at the time of sampling has been already described (Pierantoni et al., 2024).

**Table 1:** Number of bulk samples collected for each crop species at three sampling dates during the growing season.

**Date of sampling**	**Bulk samples** ***n***.			**Total**
	**March 27th**	**June 20th**	**September 6th**	
Durum wheat	4	4	4	
Common wheat	4	4	4	
Field bean	4	4	4	
Corn	4	4	4	
Tomato	4	4	4	
Muskmelon	4	4	4	
**Total**	24	24	24	**72**

A total of 72 bulk soil samples were collected across six crop species: durum wheat, common wheat, field bean, corn, tomato, and muskmelon. For each crop, four bulk samples were taken (four cores per bulk) on each of the following dates: March 27^th^, June 20^th^, and September 6^th^. This resulted in 24 samples per sampling date and an even distribution across crop species and time points.

### 2.2 DNA extraction and amplicon sequencing

Metagenomic DNA was isolated from each soil sample using the FastDNA Spin Kit for Soil (MP Biomedicals), adhering to the supplier's instructions. The DNA concentration was measured with a Nanodrop spectrophotometer (Thermo Fisher Scientific). To target the V3–V4 region of the 16S rRNA gene, PCR amplification was carried out using primers Pro341F (5′-CCTACGGGNBGCASCAG-3′) and Pro805R (5′-GACTACNVGGGTATCTAATCC-3′) (Takahashi et al., 2014). The resulting amplicons were processed into sequencing libraries by BMR Genomics (Italy) and sequenced using the Illumina MiSeq platform with paired-end 300 bp reads. All sequencing data have been deposited in the Sequence Read Archive (SRA) under the BioProject accession number PRJNA1053916.

### 2.3 Soil preparation for CE, pH, and FT-IR analyses

Soil samples were air-dried and sieved through a 2 mm mesh. A subsample of 3 g of soil was suspended in sterile distilled water in a 1:5 weight-to-volume ratio. The mixture was agitated for 2 h, followed by a 15-min settling period. The supernatant obtained was used for measuring electrical conductivity (EC), pH, and Fourier-transform infrared (FT-IR) spectroscopy. EC (1:5) was determined using an EC-Meter GLP 31 (Crison Instruments), and pH was measured with a bench-top pH meter (XS Instruments). Both parameters were measured in duplicate, and the average values were considered for subsequent statistical evaluations (Supplementary Table 1). For FT-IR spectroscopy, 30 μl of the supernatant were collected and divided into three 10 μl technical replicates, which were plated on a 384-well silica plate, following the methodology outlined by other authors (Essendoubi et al., 2005).

### 2.4 Data analysis

#### 2.4.1 Metabarcoding

All sequence preprocessing and taxonomic classification steps were performed following the standard DADA2 workflow (Callahan et al., 2016), using the *dada2* package (version 1.26.0) in R. Specifically, raw reads were filtered and trimmed based on quality profiles to remove low-quality bases and reads, including singleton handling as per DADA2 recommendations. The pipeline included error rate learning, dereplication, inference of exact sequence variants (ASVs), merging of paired-end reads, and removal of chimeric sequences. Taxonomic assignment was carried out using the naïve Bayesian classifier implemented within DADA2, with classification performed against the SILVA 138 SSU reference database (Callahan et al., 2016).

To characterize the soil microbiome diversity, both alpha and beta diversity metrics were calculated. Alpha diversity indices included the Shannon diversity index, Simpson Evenness, and Chao1 richness estimator, and the number of Observed ASVs while beta diversity was assessed using the Bray-Curtis dissimilarity index. These analyses were conducted with the *Vegan* and *microeco* packages (versions 2.7-1 and 1.15.0) in R, which provide comprehensive tools for microbial community analysis and visualization. Given that the data did not satisfy the assumptions of normality or equal variance, non-parametric statistical tests were applied to evaluate differences in relative abundance and diversity metrics. Normality was assessed using the Shapiro-Wilk test (*shapiro.test()* in R), and homoscedasticity was evaluated with the Breusch-Pagan test, performed after fitting a linear model (*lm()* function) and applying the *ncvTest()* function. This test is not a microbial-ecology-specific test but it is used as a diagnostic tool when applying regression or ANOVA-type models to microbial data.

Beta diversity patterns were visualized through Principal Coordinates Analysis (PCoA), and their statistical significance was tested using PERMANOVA. Additionally, distance-based redundancy analysis (db-RDA) was used to explore relationships between beta diversity and environmental variables, utilizing functions from *Vegan* and *microeco*. The significance of environmental variables in the db-RDA analyses was evaluated using the *cal_ordination_anova()* function implemented in the microeco R package. This method applies permutation-based testing to assess the contribution of each variable to the overall model. The core microbiome was defined by identifying ASVs present in at least 90% of samples at a minimum relative abundance threshold of 0.5%, using the *phyloseq* package (version 1.52.0). The core ASVs were subsequently analyzed statistically with the Kruskal-Wallis test. Functional potential of the prokaryotic communities was inferred using FAPROTAX via the *microeco* package, which predicts metabolic and ecological functions based on taxonomic assignments.

#### 2.4.2 FT-IR

FTIR measurements were conducted in transmission mode, with spectra recorded over the range of 4,000–400 cm^−1^ at a spectral resolution of 4 cm^−1^. For each sample, 256 scans were acquired. Spectral processing, including quality assessment, baseline correction, vector normalization, and second derivative calculation, was performed using OPUS software (version 7.5, Bruker Optics GmbH, Ettlingen, Germany). Technical replicates were averaged to obtain representative spectra, which were subsequently used for statistical analyses.

Peak picking was performed on the average raw spectra prior to derivatization, while statistical testing was carried out on the derived spectra. A wavelength-by-wavelength *t*-test (*p*-value adjusted for multiple testing using Bonferroni correction) and Principal Component Analysis (PCA) were both performed on a reconstructed spectrum, composed of the regions surrounding each identified peak, using R software, to identify significantly different spectral wavelengths and explore overall spectral variability.

## 3 Results and discussion

### 3.1 Alpha and beta diversity of the rotation crops at three sampling times

Being six distinct species, the rotation crops exhibited distinct growth stages at the three sampling times. In March, winter crops (field beans, soft wheat, and durum wheat) were green and actively growing, while summer crops (tomato, corn, and muskmelon) had not been established yet, thus the soil was bare. In June, winter crops had reached maturity and showed dry vegetation, while summer crops were in their full vegetative stage. Finally, in September, winter crops and muskmelon had been harvested, leaving the soil with just the straw mulch. Corn and tomato were in the maturation stage, with dry vegetation and some remaining fruit on tomato plants (Supplementary Figure 1).

In order to assess the variations of soil microbiota as a function of the crop stage and of the specific crops, both alpha and beta diversity were compared between bare soil and soil with vegetative plants and among the six crops, for all the three sampling times, as detailed below.

**Figure 1:**
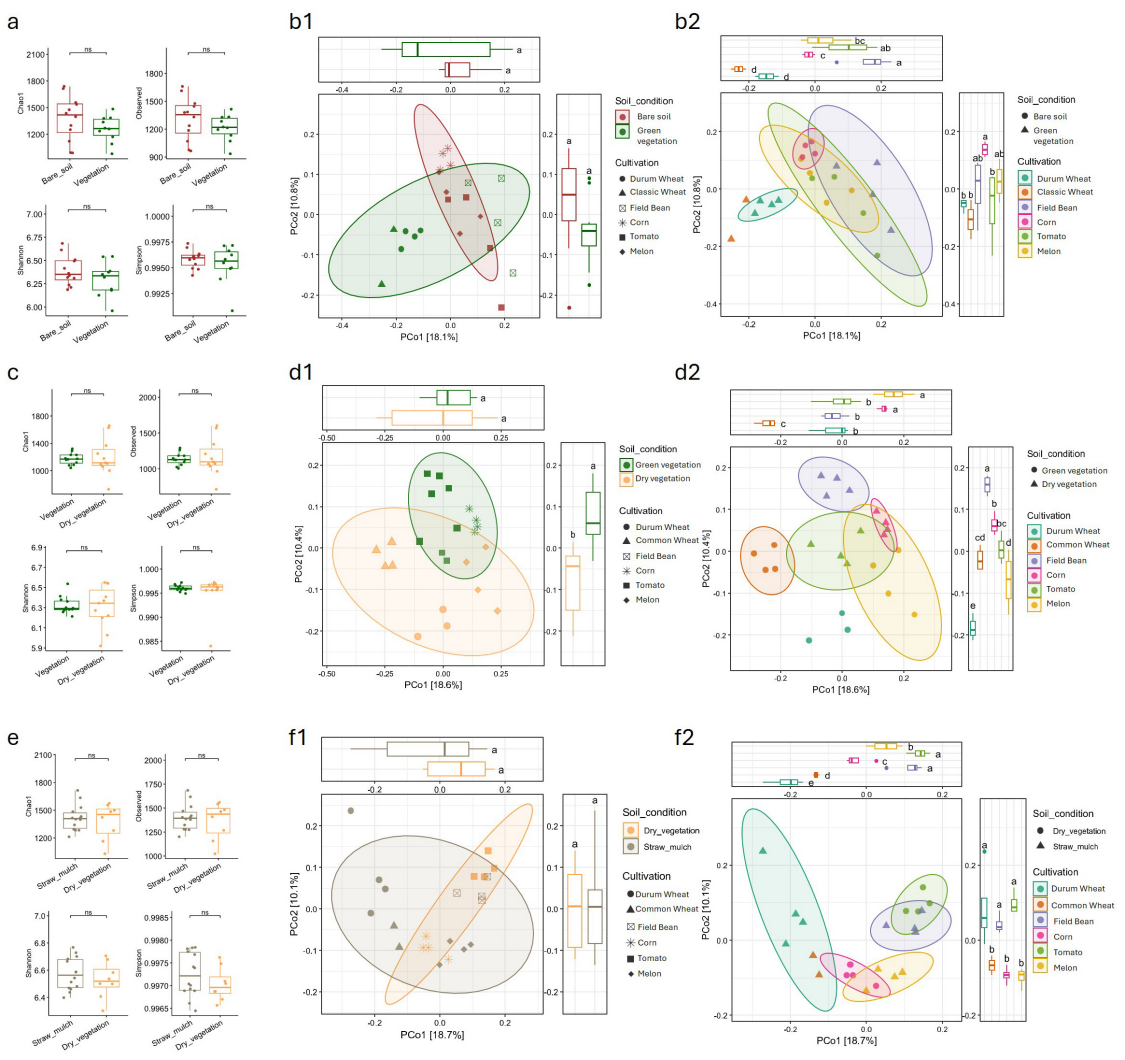
Alpha and beta diversity of soil microbial communities across different seasons (March, June, September) and crop cultivations. **(a, c, e)** Boxplots show alpha diversity indices, for each crop and season, grouped by cultivation stage (soil condition), with statistical comparisons between groups. **(b1, d1, f1)** Principal Coordinate Analysis (PCoA) plots based on Bray-Curtis dissimilarity illustrate beta diversity by soil condition for each sampling, with 95% confidence ellipses. **(b2, d2, f2)** PCoA plots further differentiate beta diversity by crop type within each season, with corresponding boxplots for PCoA axes. Distinct clustering patterns and diversity metrics highlight the influence of both seasonality and crop type on soil microbial community structure. Statistical significance is indicated where relevant.

In March, none of the alpha diversity metrics revealed statistically significant differences between the microbiota of bare soil plots and those with green vegetation, that is, between plots with summer and winter crops (Figure 1a); similarly, no significant differences were observed among the individual rotation crops (Supplementary Figure 2a). A similar outcome was observed for beta diversity in the PCoA diagram, based on Bray-Curtis index, where no spatial separation between growth stage variable was detectable. When considering the single crops as a variable, PCoA showed that on PCo1 DW and CW clustered closely together while Field Bean and summer crops overlapped, with Tomato being the most spread out (Figures 1, b1, b2).

In June, alpha diversity did not show any significant differences between fields with green vegetation (summer crops) and those with dry vegetation (winter crops), as among the single crops (Figure 1c and Supplementary Figure 2b). Beta diversity analysis revealed some clustering based on the vegetation stage, particularly along PCo 2 (Figures 1, d1), where the two groups were clearly separated.

When considering the crop variable, more distinct clusters emerged compared to the March sampling. Winter crops, along with melon, formed separate clusters, while corn and tomato showed slight overlap with the field bean cluster (Figures 1, d2).

Finally, the September sampling resembled that of March, showing no significant differences in alpha diversity (Figure 1e, Supplementary Figure 2c) as well as no difference in terms of beta diversity were the straw mulch samples (winter crops and melon) overlapped with the dry vegetation samples (corn and tomato) on the PCoA diagram (Figures 1, f1). Differently from the March sampling, when combining the PCoA analysis with the crop species variable, three main groupings were observed: durum wheat and soft wheat clustered together, as did corn and melon (despite being at different growth stages), and tomato and field bean (also at different growth stages; Figures 1, f2). When considering all samples from the different sampling points and sampling times, a clear grouping is observed based on the sampling time. The September samples are tightly clustered together, distinctly separated from the June and March samples, which instead overlap (Supplementary Figure 3).

The results suggest that crop growth stage and crop species have a relatively modest and context-dependent impact on soil microbial diversity and composition within the studied rotation system. Across all three sampling periods, alpha diversity showed no significant differences between bare soil and vegetated plots or among the six crop species. This indicates that the presence of vegetation, as well as the specific crop, did not consistently affect the overall richness or evenness of the soil microbial community. These findings are in line with recent studies showing that while plant species can influence soil microbiota, their effects are often outweighed by factors like soil type, season or other environmental conditions (Wagg et al., 2014; Prober et al., 2015; Hartman et al., 2017). The fact that in most cases the samplings were obtained from soil between rows, and therefore relatively far from plant roots, further enhances the observation that the microbiota of bare soil is not significantly different from that of soil with growing crops (Peiffer et al., 2013; Zhalnina et al., 2018). At the same time, this observation within the normal rotation is not necessarily extensible to agronomic practices consisting in leaving the soil without crops for long periods of time.

### 3.2 Beta diversity and soil physio-chemical parameters variation across crop rotation and sampling time

**Figure 2:**
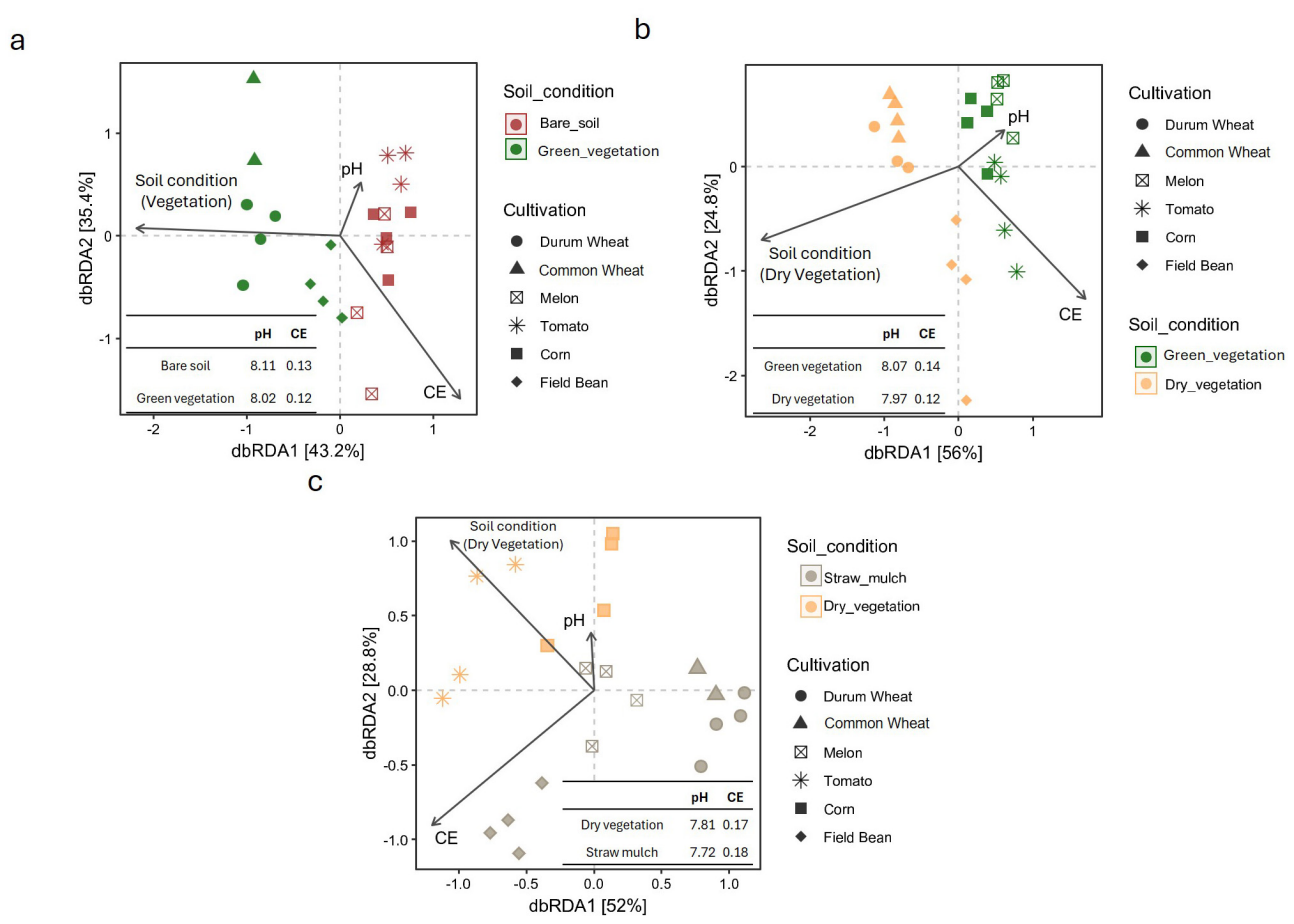
Distance-based redundancy analysis (dbrda) biplots illustrating the relationships between soil cover types and physicochemical properties (pH and electrical conductivity, CE) across different crop species, for each sampling time [March, panel **(a)**; June, panel **(b)**; September, panel **(c)**]. Different symbols represent crop types (e.g., Durum wheat, Common wheat, Muskmelon, Tomato, Corn, Field bean), and arrows indicate the direction and strength of soil variables' influence. Inserts show mean pH and CE values for each soil condition.

The relationship between soil beta diversity and physico-chemical factors, specifically pH and electrical conductivity (EC), was examined using distance-based redundancy analysis (dbRDA) (Figure 2). In March, the first two dbRDA axes (dbRDA1 and dbRDA2) together explained 78.6% of the total variation (Figure 2a). pH was positively associated with dbRDA1, while EC showed a positive correlation with dbRDA2. These environmental variables influenced the clustering of soil communities, particularly in field sections where winter crops were present at the time of sampling. In these areas, samples are clustered closely by crop species. In contrast, samples from unplanted fields intended for summer crops (i.e., bare soil) displayed weaker clustering and greater overlap, mainly along the dbRDA1 axis. Overall, soil conditions, reflected by pH (no significance with permutation test) and EC (permutation test *p*-value = 0.099, weak significance), appear to be key drivers of community structure (permutation test *p*-value = 0.012), with samples from vegetated fields (winter crops) clearly separating from bare soil samples (summer crops) along dbRDA1. This pattern was further confirmed by a permutational multivariate analysis of variance (PERMANOVA), which resulted in a significant *p*-value = 0.027.

In June, when all fields were covered either with green vegetation (summer crops) or dry biomass (winter crops), a distinct separation was observed along the first dbRDA axis (PERMANOVA, *p*-value = 0.009, permutation test *p*-value = 0.012), which accounted for 56% of the total variation (Figure 2b). Common and durum wheat samples clustered closely together, while field bean samples were more widely distributed along the second axis (dbRDA2), which explained an additional 24.8% of the variation. By September, sample dispersion increased across the ordination space (Figure 2c). Soil samples from each field, four of which now contained straw mulch with no active crops, formed distinct groupings, with only the wheat-associated samples remaining tightly clustered. Soil conditions continued to drive sample separation, with a diagonal gradient visible across the dbRDA but at this sampling time this spatial separation was not supported by a statistical significance (PERMANOVA, *p*-value = 0.129, permutation test *p*-value = 0.065).

The pH and electrical conductivity (EC) variables, represented as vectors in the dbRDA plots, are summarized in each panel of Figure 2. Across all sampling periods (March, June, and September), pH consistently appeared as a short vector, indicating a relatively weak influence (no statistical significance with permutation test) on microbial community structure, an observation supported by the lack of statistically significant differences in pH among crop types. In contrast, EC was consistently represented by longer vectors, suggesting a stronger impact on microbial composition. This is further supported by a greater number of significant differences in EC, particularly in September, when tomato and field bean samples showed the most pronounced contrasts (e.g., Tomato vs. DW, CW, and C; Field bean vs. DW, CW, and C) and by a significant *p*-value obtained via permutation test (0.34).

Finally, when combining all the samples in the same dbRDA analysis, two distinct separation patterns emerge. In Supplementary Figure 4a, the physio-chemical parameters were considered alongside the sampling time and crop variable. Two main separations are evident: along the dbRDA1 axis the crop variable plays a dominant role here (permutation test *p*-value = 0.001), with the CW vector aligning almost parallel to the axis, pointing to the left, while the field bean vector points in the opposite direction. Notably, the wheat samples are clustered on the far left of the plot, indicating a strong differentiation between wheat and the other crops. This suggests that the crop type influences the variation in physio-chemical parameters along this axis. Along the dbRDA2 axis the separation is primarily driven by sampling time (permutation test *p*-value = 0.001). The September samples are distinctly grouped toward the lower portion of the plot, ie, separating the CW and DW soil communities sampled in March and June from those sampled in September. This indicates that time of sampling has a strong influence, with a clear distinction between samples taken at different times (September vs. March and June). The effect of sampling time may reflect seasonal changes, such as differences in environmental conditions, plant growth stages, or other temporal factors.

For this reason, the dbRDA was obtained considering the same data but introducing the soil condition parameter along with the sampling time (Supplementary Figure 4b). This change resulted in better grouping of wheat samples (CW and DW), which are here more clearly clustered in the upper part of the plot (still partially separated by the sampling time variable). Along the dbRDA1 axis, the most noticeable separation is between September samples (Dry Vegetation and Straw Mulch, the latter only present at this time) and those from March and June (Bare Soil and Green Vegetation, which overlap).

The crop species and sampling time are the main factors driving this clustering (no significance for the Soil_condition variable with permutation test). Specifically, samples with dry vegetation are scattered throughout the plot, with DW and CW (June) separated from Field Bean (June) and Tomato and Melon (September). Straw mulch samples overlap with dry vegetation samples from the same sampling time. The crop variable acts as a strong driver as well, with wheat samples (March and June) grouped together, while Tomato and Melon form a tight cluster (March and June), along with Field Bean and Tomato.

Beta diversity analyses revealed clearer patterns than alpha diversity in this crop rotation system. In both March and September, microbial communities showed little differentiation based on vegetation stage or crop species, suggesting substantial overlap regardless of whether vegetation was present. In contrast, June samples displayed more distinct clustering by crop type, which coincided with the period when the difference between dry winter crops and green summer crops was most pronounced.

The limited impact of crop species and the absence of significant differences in alpha diversity further suggest that soil physico-chemical properties and the existing microbial community play a stronger role in shaping soil microbiota than plant identity alone (Fierer and Jackson, 2006; Lauber et al., 2009). Seasonal factors, including fluctuations in soil moisture, temperature, and organic matter inputs from crop residues, appear to be key drivers of the temporal clustering observed, particularly the clear separation seen in September samples (Berg and Smalla, 2009; Siles and Margesin, 2017).

Distance-based redundancy analysis (dbRDA) reinforced the importance of soil properties, with electrical conductivity (EC) emerging as a stronger driver of microbial beta diversity than pH across sampling periods (Lauber et al., 2009; Rousk et al., 2010). The significant separation between vegetated plots (winter crops) and bare soil plots (summer crops) in March highlights the role of root activity and plant presence in shaping microbial communities. Crop-specific clustering within vegetated plots is consistent with previous research showing that different plant species can influence rhizosphere microbial composition, while bare soil plots tended to show more uniform microbial profiles likely shaped by abiotic factors (Bulgarelli et al., 2013; Hartman et al., 2017).

In June, the dbRDA showed clear shifts in microbial community structure linked to both crop species and growth stage. Field bean, in particular, displayed greater microbial heterogeneity, likely reflecting the effects of nitrogen fixation and associated changes in soil nutrient dynamics (Mendes et al., 2015; Goss-Souza et al., 2017). By September, microbial communities were more dispersed, with distinct groupings especially noticeable in plots with straw mulch and no active crops. This pattern likely reflects the influence of residue decomposition and environmental variability on soil microbial structure (Sun et al., 2015; Kavamura et al., 2019). The continued clustering of wheat-associated samples suggests that some crop-specific microbial signatures can persist, even though linked to seasonal transitions (Schlatter et al., 2017).

Overall, these results highlight the complex interplay between soil properties, crop identity, and seasonal dynamics in shaping soil microbial communities. While plant species and vegetation stage can influence microbial structure, their effects are often temporary and appear to be outweighed by soil characteristics and broader environmental conditions over the course of the crop rotation (Fox et al., 2022; Frene et al., 2024).

### 3.3 Taxonomic profiling of dominant genera across crop species, crop growth stage and sampling time

**Figure 3:**
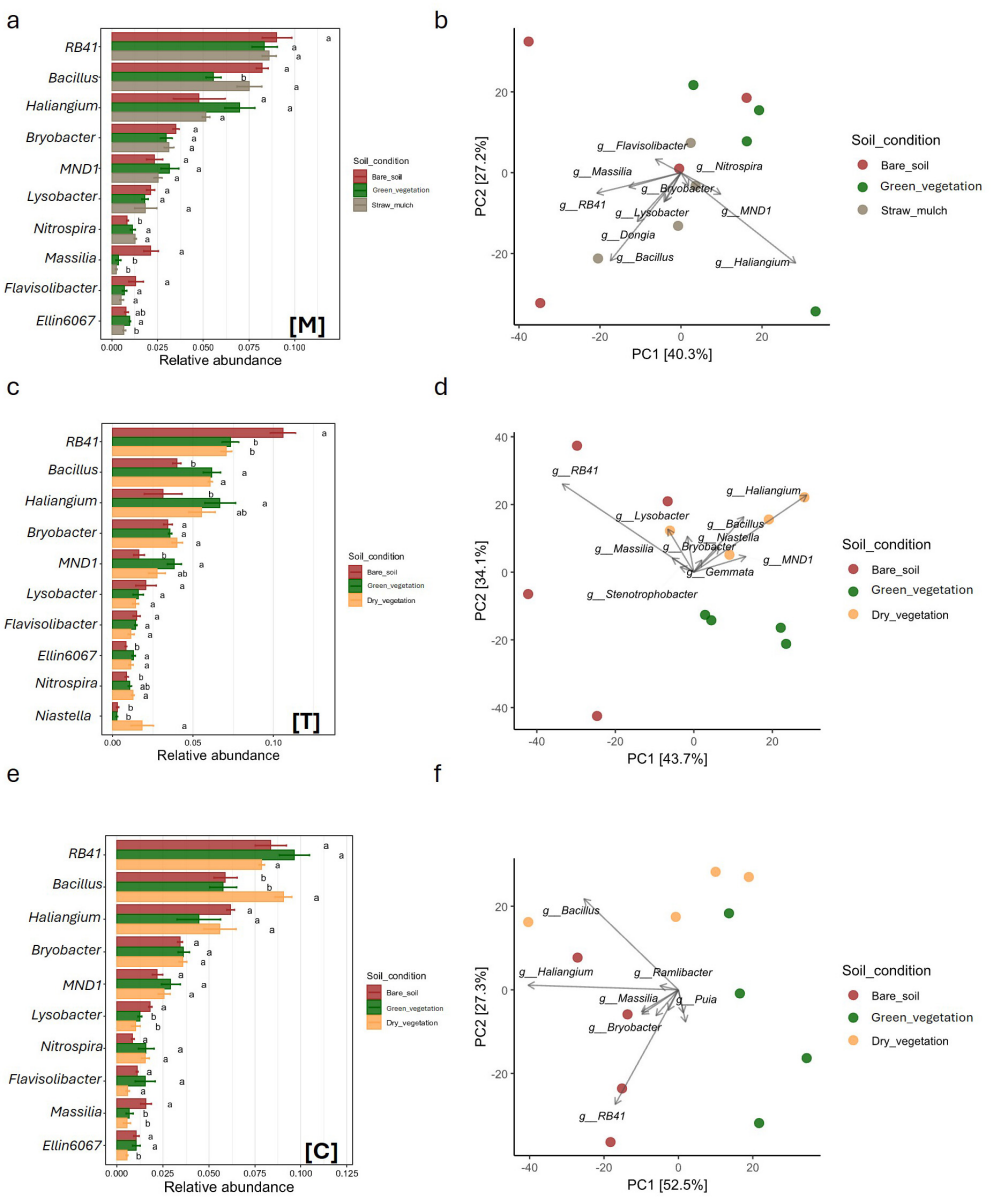
Comparison of soil bacterial community structure under three distinct soil condition, green vegetation (green), dry vegetation (yellow), and straw mulch (gray), across three crop types: Durum Wheat [Dw], Common Wheat [Cw], and Field Bean [Fb]. Panels **(a, c, e)** display bar plots of relative abundances for dominant bacterial genera, with statistical significance among treatments indicated by different letters above bars **(a, b, ab)**. Panels **(d, e, f)** present PCA ordinations depicting the variation in bacterial community composition based on genus-level data, with samples color-coded by soil condition. Axes denote principal components PC1 and PC2, alongside the percentage of variance explained. Directional vectors highlight bacterial genera contributing to separation among treatments.

**Figure 4:**
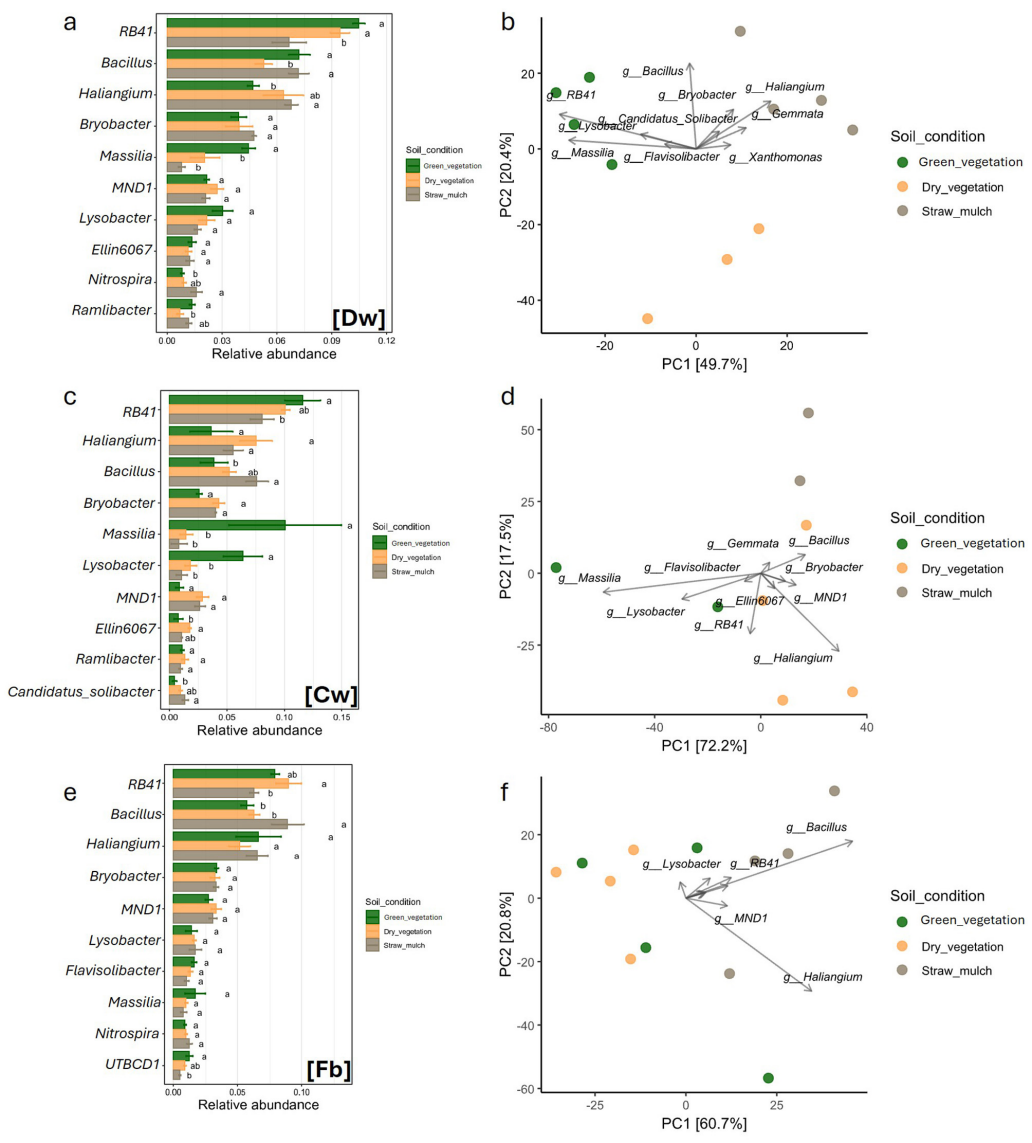
Comparison of soil bacterial community structure under three distinct soil condition, bare soil (brown), dry vegetation (yellow), and straw mulch (gray), across three crop types: Muskmelon [M], Tomato [T], and Corn [C]. Panels **(a, c, e)** display bar plots of relative abundances for dominant bacterial genera, with statistical significance among treatments indicated by different letters above bars **(a, b, ab)**. Panels **(d, e, f)** present PCA ordinations depicting the variation in bacterial community composition based on genus-level data, with samples color-coded by soil condition. Axes denote principal components PC1 and PC2, alongside the percentage of variance explained. Directional vectors highlight bacterial genera contributing to separation among treatments.

Six genera ranked among the top ten across all crop species and sampling times (Figures 3a, c, e, 4a, c, e). Specifically, three genera belonged to the *Actinobacteriota* phylum: *Haliangium, Bryobacter*, and *Lysobacter*. Two genera belonged to the *Proteobacteriota* phylum: *RB41* and *MND1*, while one genus, *Bacillus*, belonged to the *Bacillota* phylum. Three genera were present in all microbial communities except one. *Massilia* (*Proteobacteriota*) was absent from the top 10 genera in the tomato samples, *Ellin* (*Actinobacteriota*) was missing from the top 10 in the field bean sample, and *Nitrospira* (*Nitrospirae*) was not found among the top 10 in the Common Wheat samples. Other genera present in the top ten were *Ramlibacter* (phylum *Actinobacteriota*; only present in DW and CW samples), *Solibacter* (*Acidobacteriota*; CW samples), *Flavisolibacter* (*Acidobacteriota*; absent from CW and DW), UTBCD1 (only present in FB samples) and *Niastella* (Actinobacteriota; only present in tomato samples).

Among the winter crops, the three sampling times in DW formed distinct clusters in the PCA plot, which explained 70.1% of the total variability (Figures 3b, d). These clusters were primarily separated by four key taxa: *Massilia, RB41, Bacillus*, and *Haliangium*. In CW, where the separation between sampling times was less pronounced despite a higher explained variability (89.7%), *Haliangium* and *Bacillus* emerged as the main drivers. Finally, in field bean, the PCA plot (81.5% explained variability) did not reveal clear clustering among sampling times, but *Bacillus* and *Haliangium* were again the dominant taxa, as observed in CW. Among the summer crops, none showed distinct groupings in the PCA plot based on taxonomic relative abundances (Figures 4b, d, f). Melon samples were broadly scattered across the PCA space (67.5% of total variability explained), with *Haliangium* and *Bacillus* as the main contributing taxa. In tomato, the June (green vegetation) and September (dry vegetation) samples clustered together, while the March samples (bare soil) were more scattered. Key drivers included *RB41, Bacillus*, and *Haliangium* (77.8% explained variability). For corn, the March and June samples (bare soil and green vegetation, respectively) were clearly separated along the first principal component (PC1), while the September samples partially overlapped with both time points (79.8% explained variability). Overall, the taxonomic analysis revealed that several genera from Actinobacteriota (*Haliangium, Bryobacter, Lysobacter*), *Proteobacteriota* (RB41, MND1), and *Bacillota* (*Bacillus*) dominate across crops and sampling times. These taxa are known for their diverse ecological roles, including organic matter decomposition, nutrient cycling, and plant growth promotion (Glick, 2012; Mendes et al., 2015). The presence of crop-specific taxa, such as *Ramlibacter* in wheat and *Niastella* in tomato, suggests niche specialization, possibly driven by root exudate composition or soil microenvironmental conditions (Peiffer et al., 2013; Zhalnina et al., 2018). The PCA clustering patterns further support that microbial community composition varies with crop species and sampling time, with winter crops showing stronger temporal differentiation than summer crops (Philippot et al., 2013; Chaparro et al., 2014).

### 3.4 Temporal shifts in predicted nitrification activity across crops

Functional Annotation of Prokaryotic Taxa (FAPROTAX) was used to predict functional profiles of the prokaryotic communities of the soil samples, based on taxonomic assignments from sequencing data. In addition to providing overall community-level functional predictions, FAPROTAX enables the inference of specific traits and functions associated with individual taxa. Taxonomic profiles were mapped against the FAPROTAX database to infer putative biogeochemical functions of the detected prokaryotes. Thus, inferred functions represent putative capabilities rather than observed metabolic functions *in situ*.

**Figure 5:**
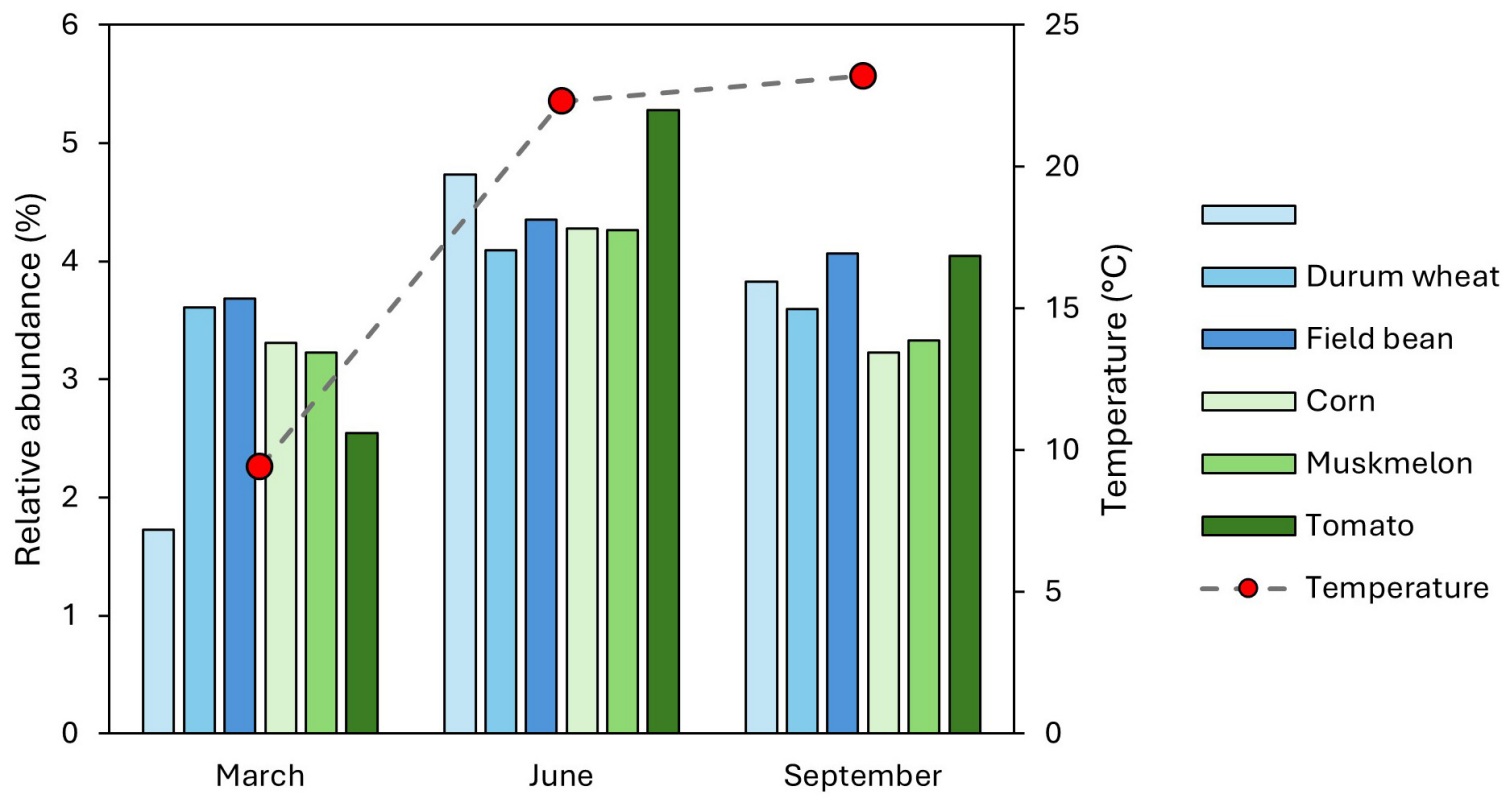
Temporal trends in nitrification profile abundances across rotation crops (common wheat, durum wheat, field bean, corn, muskmelon, and tomato) measured at three sampling times: March, June, and September. Bar plots indicate the percentage of nitrification profiles per crop (left *y-*axis), with temperature data (right *y*-axis) overlaid to illustrate environmental context.

Among the predicted functional profiles, those associated with nitrification were selected for more detailed analysis (Figure 5). All crops were analyzed across the three sampling times (March, June, and September). Figure 5 illustrates the percentage of nitrification profiles for each crop over the specified months. Notably, all crops exhibited relative abundances of nitrification profiles that peaked in June.

All winter crops showed a significant increase in nitrification profiles in June compared to March and September. Corn, Muskmelon, and Tomato also demonstrated a peak in June, indicating a seasonal trend in nitrification activity across different crops. This pattern suggests that environmental factors, such as temperature, which peaks in the warm months (June and September) may influence the nitrification processes in soil more than the crop type.

Functional predictions using FAPROTAX highlighted nitrification-related functions as prominent across samples, reflecting the importance of nitrogen cycling in these agricultural soils (Louca et al., 2016, 2018). This is consistent with the known roles of dominant taxa such as *Nitrospira* and *Bacillus* in nitrification (Glick, 2012; Daims et al., 2015). The variation in nitrification potential among crops and sampling times may have implications for soil fertility management, warranting further investigation (Philippot et al., 2009; Butterbach-Bahl et al., 2013).

### 3.5 Core microbiota variation among crops

ASVs present in more than 90% of samples within each crop type and sampling month, and with a minimum relative abundance of 0.5%, were defined as the core microbiome. To assess the influence of crop type within each sampling month, variations in these core communities were analyzed after grouping the ASVs at the genus level, allowing for the detection of major compositional shifts.

**Figure 6:**
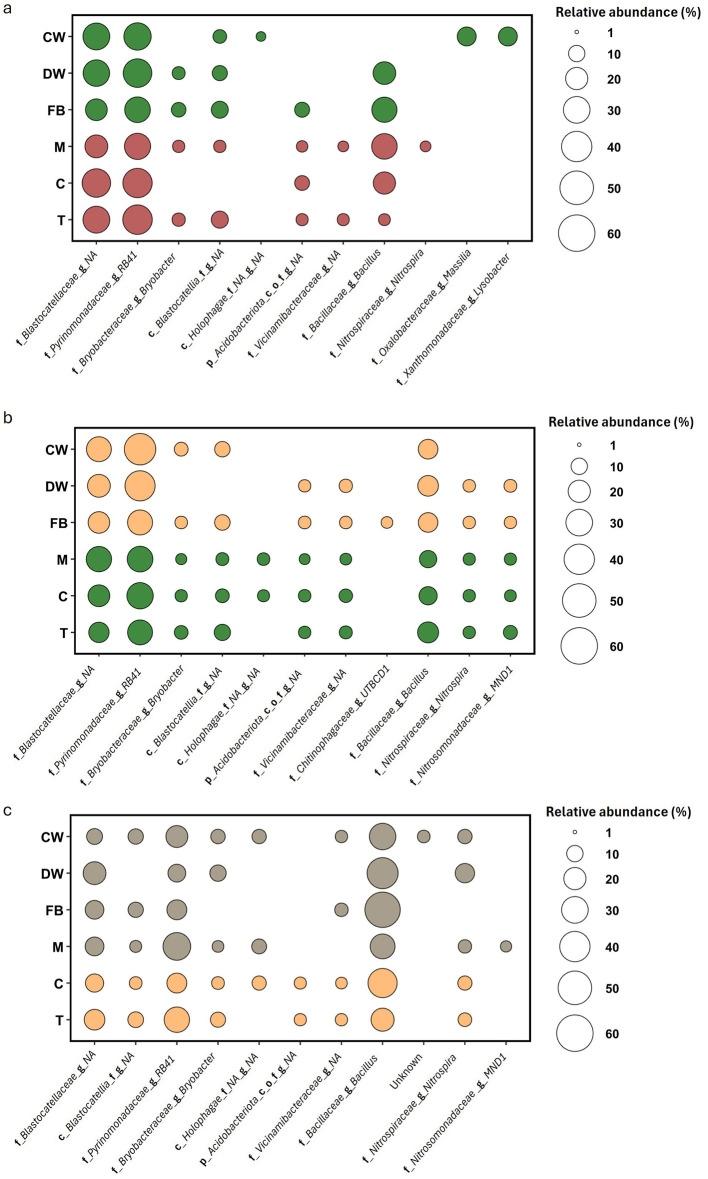
Core microbiota composition of the six crops, common wheat (CW), durum wheat (DW), field bean (FB), corn (C), tomato (T), and muskmelon (M), across the three seasonal sampling times: March **(a)**, June **(b)**, and September **(c)**. Each panel displays bubble plots depicting the relative abundance of dominant bacterial taxa per crop, with bubble size reflecting proportional abundance. Crop stages are represented by color: brown = bare soil, yellow = dry vegetation, green = green vegetation, and gray = straw mulch.

In March, a total of 11 genera (21 ASVs) were identified as part of the core microbiota across all rotation crops (Figure 6a). Specifically, the core microbiota of CW and FB each included six genera, while DW had 5. Among the summer crops, which had bare soil at the time of sampling, Melon exhibited the highest number of core genera with 8, followed by Tomato with 7, and Corn with only 4. Only two genera, both belonging to the Acidobacteriota phylum and exhibiting the highest relative abundance among the core genera, were present in all rotation crops. At the time of sampling, the core microbiota of DW and FB were very similar, differing by only one genus. The same was true for Melon (M) and Tomato (T), which also differed by just one genus in their cores. Overall, the core microbiota was primarily composed of genera from the Acidobacteriota phylum, followed by *Bacillus*. Notably, only CW included two genera from the Proteobacteria phylum (*Massilia* and *Lysobacter*), while *Nitrospira* was found exclusively in Melon.

In June, the summer crops with green vegetation exhibited nearly identical core microbiota, except for tomato, which had one fewer genus than the others (Figure 6b). Specifically, the cores of Melon (M) and Corn (C) each contained 10 genera, with seven belonging to the Acidobacteriota phylum, while tomato's core included six genera from this phylum. The remaining three core genera, *Bacillus, Nitrospira*, and *MND1*, were consistent across all three crops. Greater variation was observed among the winter crops, which had dry vegetation at the time of sampling. Specifically, only three genera, *RB41*, an unknown genus from the *Blastocatellaceae* family, and *Bacillus*, were shared by all three rotation crops. Among them, DW and FB shared a larger number of genera (7), indicating a closer similarity. Overall, in June the six crops shared three genera, similar to March, with those belonging to the *Acidobacteriota* phylum being the most abundant, followed by *Bacillus*.

In September, the same three core genera remained the most abundant, but with a shift in dominance toward *Bacillus*, which became the most prevalent genus (Figure 6c).

Corn (C) and Tomato (T), the only remaining crops with vegetation, exhibited similar core microbiota, differing by just one genus out of nine. The four crops with straw mulch shared some core genera sporadically, but no clear pattern or trend was evident.

The core microbiota remained largely stable across all sampling times, showing only minor variations between rotation crops and predominantly the same genera present. Notable exceptions include Massilia and *Lysobacter*, found only in March in CW; the genus UTBCD1 (from the *Chitinophagaceae* family), present exclusively in June in Field bean; and the genus MND1 (*Nitrosomonadaceae* family), detected in June across all crops except CW, and in September only in Melon.

This study identified a stable core microbiome shared across different crop species and sampling periods within the rotation system. This core community was dominated by genera belonging to the Acidobacteriota phylum and the genus *Bacillus* (Bacillota), which were consistently present in over 90% of samples and showed notable relative abundance. The persistence of these groups points to a resilient microbial consortium likely playing key roles in essential soil functions, such as nutrient cycling and organic matter breakdown (Fierer, 2017; Jiao et al., 2019). The strong presence of Acidobacteriota aligns with their well-documented ability to thrive under a wide range of soil conditions, while the spore-forming capacity of *Bacillus* species may help them persist through periods of environmental stress or during the decomposition of crop residues (Nicholson et al., 2000).

Interestingly, whilst *Bacillus* is considered a r-strategist (Stone et al., 2023), Acidobacteriota have been demonstrated to be K-strategists (Li et al., 2025), suggesting that the latter paly a constant role while the former exert their metabolic activity in specific moments during the years. The interplay between bacteria with different life history strategies, or K/r selection, would ensure a long term coexistence of different bacteria adapted to low-nutrient (K-strategist) or nutrient-rich (r-strategists) conditions (Yin et al., 2022). The variations of vegetation and the micro-niches represented by the soil close or far away from the roots are likely to represent this nutrient variability and explain the presence of Acidobacteria and *Bacillus* in the core microbiota of the studied soils.

Although the core microbiome was broadly consistent, minor differences in core genera were observed between crops and sampling times, reflecting subtle but potentially meaningful shifts in microbial composition linked to plant species or growth stage. For instance, the presence of *Massilia* and *Lysobacter* exclusively in wheat plots in March, and *UTBCD1* in field bean plots in June, likely reflects crop-specific influences such as differences in root exudates or soil microhabitat conditions (Zhalnina et al., 2018). Similarly, the detection of *Nitrospira* as part of melon's core microbiome points to possible crop-specific impacts on nitrogen cycling, highlighting the functional implications of plant-microbe interactions (Daims et al., 2015).

The increase in *Bacillus* abundance observed in September coincided with the buildup of straw mulch and senescing vegetation, supporting the idea that spore-forming, stress-tolerant taxa play a key role in residue decomposition and nutrient turnover during the post-harvest period.

### 3.6 FTIR-based soil profiling

Fourier Transform Infrared Spectroscopy (FTIR) was performed on soil samples from all crops at each sampling time. Seventeen characteristic peaks (p1–p17) were identified, and the analysis was restricted to the wavelength intervals corresponding to these peaks.

**Figure 7:**
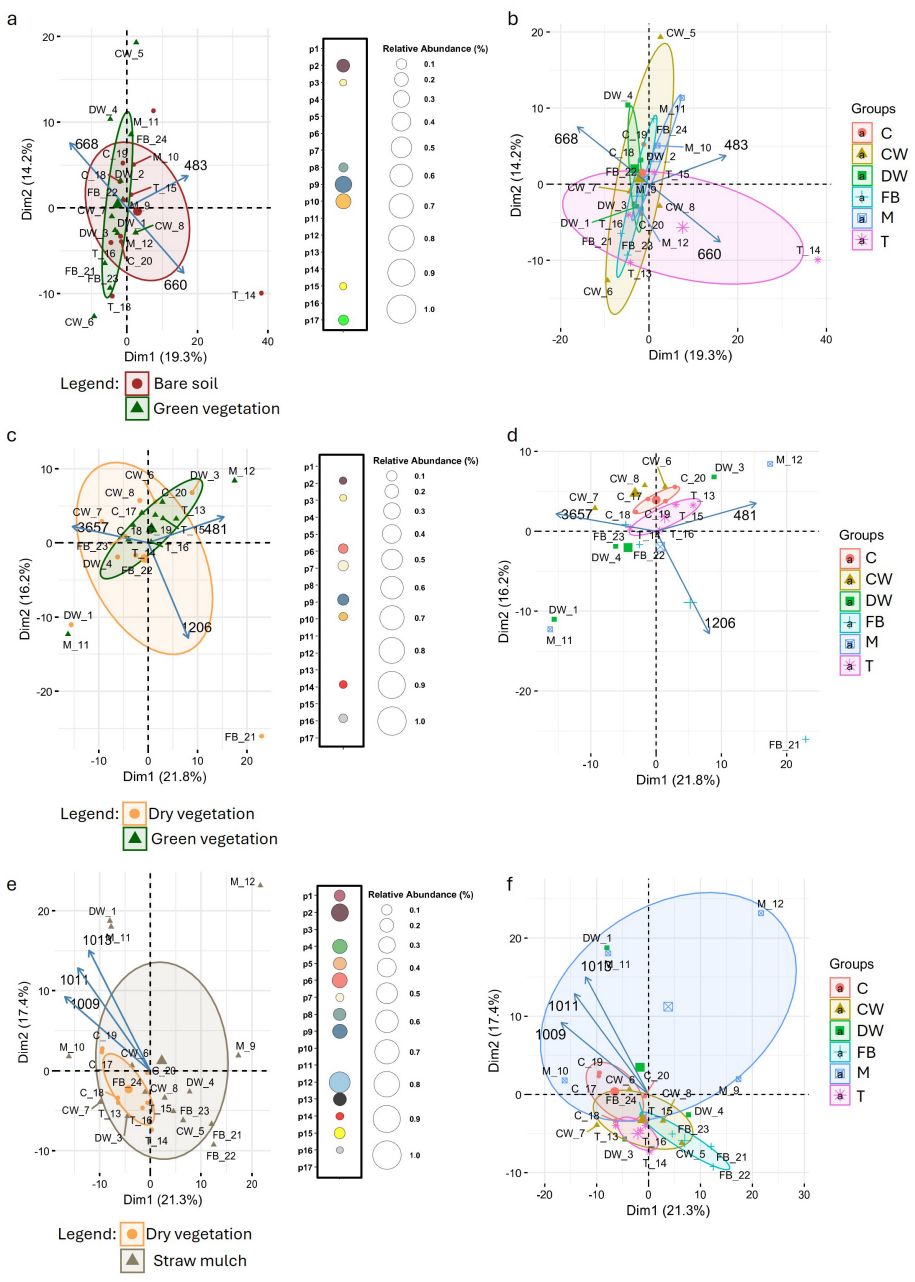
FTIR-based characterization of soil spectral variation across crop stages and species over three sampling periods: March [panel **(a)** and **(b)**], June (panel **(c)** and **(d)**], and September [panel **(e)** and **(f)**]. PCA biplots illustrate sample clustering based on FTIR spectral profiles, color-coded by crop stage (bare soil, dry vegetation, green vegetation, straw mulch) in panels a, c, and d, and by crop species, Common Wheat (CW), Durum Wheat (DW), Field Bean (FB), Corn (C), Tomato (T), and Muskmelon (M), in panels **(b, d, e)**. PCA loadings highlight key wavelengths driving separation among conditions (crop growth stage or crop species). Adjacent bubble plots [panels **(a, c, e)**] visualize the number of significantly different spectral wavelengths, for each peak (p1–p17) across crop stages, derived from *t*-tests, with bubble size representing the relative spectral abundance.

To explore the influence of vegetation stage and crop species on soil spectral profiles, PCA was applied to the FTIR data and visualized as biplots (Figure 7). The biplots displays the three principal loadings corresponding to the wavelengths that contributed most strongly to the separation along the first two principal components. In March (Figures 7a, b), PCA did not reveal a clear separation of samples according to vegetation stage. A wavelength-specific *t*-test comparison between vegetation stages identified only a limited number of wavelengths, belonging to seven different peaks, showing statistically significant differences (p2–3, p8–10, p15 and p17). Likewise, PCA performed with crop type as the grouping factor showed no clear separation among samples in March. At this sampling time, the wavelengths that contributed most strongly to the separation along the first two principal components, specifically 668, 483, and 660 cm^−1^. These wavelengths belong to peaks p14, p16, and p15, respectively, all of which are associated with mineral signals. This pattern was further confirmed by a permutational multivariate analysis of variance (adonis2), which indicated that neither crop type (*p* = 0.112) nor vegetation stage (*p* = 0.089) had a statistically significant effect on the soil spectral profiles.

In June (Figures 7c, d), PCA again showed no clear sample separation based on vegetation stage. Adonis2 confirmed the absence of significant effects, with *p*-values of 0.1 for crop type and 0.791 for vegetation stage. The three principal loadings for the June PCA corresponded to wavelengths 3,657, 481, and 1,206 cm^−1^, belonging to peaks p1, p16, and p3, respectively. Peak p1 is attributed to mineral signals, specifically free –OH groups from clays and silanol groups, with no likely microbial origin. Peak p16 is associated with silicates and quartz (mineral signal), while peak p3 corresponds to microbial/organic signals, including C–O and C–N stretching, carbohydrates, phosphates, and possible microbial polysaccharides. A wavelength-specific *t*-test for June revealed a limited number of significant wavelengths, belonging to peaks p2, p3, p6, p7, p9, p10, p14, and p16. Among these, peaks p2, p9, p10, p14, and p16 are of mineral origin; peak p3 is associated with carbohydrates of microbial or organic origin; and peaks p6 and p7 are linked to both organic and mineral signals.

In September (Figures 7e, f), PCA again did not reveal any clear separation of samples based on either vegetation stage or crop type. This was further confirmed by adonis2, which showed no statistically significant effects for either factor. The three principal loadings for the September PCA corresponded to wavelengths 1,009, 1,011, and 1,013 cm^−1^, all belonging to peak p7. This peak is associated with both organic and mineral signals, reflecting a strong silicate contribution but with possible microbial polysaccharides. Interestingly, September was the month with the highest number of significant wavelengths detected by *t*-test comparisons. Only peaks p3, p10, p11, and p17 did not show any significant wavelengths.

Overall, the soil metabolome, as assessed through FTIR, proved to be a good indicator of differences between samples collected at different time points, particularly between March and September (PERMANOVA, *p*-value = 0.042). In contrast, this approach was not effective in detecting differences related to crop type or vegetation stage, as both PCA and PERMANOVA analyses consistently showed no clear separation or statistically significant effects for these factors. Furthermore, the differences observed among crops and vegetation stages through metabarcoding analysis were not reflected in the metabolome profiles obtained by FTIR, suggesting that microbial community shifts do not necessarily translate into detectable changes in the bulk soil chemical fingerprint captured by this technique.

In addition to microbial community analysis, Fourier Transform Infrared Spectroscopy (FTIR) provided insight into soil chemical profiles, showing that sampling time had a stronger influence than crop type or vegetation stage. The clear differences observed between March and September samples likely reflect seasonal changes in organic matter inputs and microbial activity, which can affect both the quantity and composition of soil organic compounds, as well as mineral-organic interactions (Baldock and Skjemstad, 2000; Kögel-Knabner, 2002). In contrast, FTIR did not reveal consistent separation based on crop type or vegetation stage, suggesting that bulk soil chemical properties remain relatively stable over short timescales and are largely shaped by the mineral matrix and persistent organic matter pools. This supports previous findings that shifts in microbial community composition, while detectable with high-resolution sequencing, do not necessarily lead to immediate or large-scale changes in the soil's chemical signature detectable by FTIR (De Mastro et al., 2019, 2020).

The difference between microbial and chemical soil patterns highlights the value of combining molecular and chemical approaches. While metabarcoding captures subtle taxonomic and functional changes in microbial communities, FTIR reflects broader, integrated chemical characteristics of the soil, which tend to shift more gradually and under the influence of multiple biotic and abiotic factors. Looking ahead, applying more sensitive metabolomic techniques, such as nuclear magnetic resonance (NMR) spectroscopy or mass spectrometry, could provide deeper insights into the biochemical consequences of microbial dynamics in soil systems (Viant, 2008).

Together, these findings point to the presence of a stable core microbial community that persists across crops and seasons, likely contributing to functional stability in the soil. At the same time, the crop-specific and seasonal variations in certain microbial groups suggest opportunities for targeted microbiome management to support soil fertility and crop performance. The strong influence of seasonal factors on soil chemical properties further underscores the importance of considering temporal variation when assessing soil health in agricultural systems.

## 4 Conclusions

This study shows that in the examined crop rotation system, soil microbial diversity and community composition are driven more by soil properties and seasonal changes than by crop species or growth stage. While plants can cause short-term shifts in microbial communities, these effects are generally modest and temporary. A stable core microbiome persists across crops and seasons, likely supporting key soil functions such as nutrient cycling and organic matter decomposition.

Seasonal factors, including residue decomposition and environmental variability, strongly influence both microbial communities and soil chemistry, highlighting the importance of considering temporal dynamics in soil management.

Future research should focus on finer-scale sampling (e.g., rhizosphere vs. bulk soil), incorporate functional profiling through metagenomics or metatranscriptomics, and assess soil physicochemical variables to elucidate the mechanisms driving microbial dynamics. Additionally, understanding the impact of crop residues and mulching on microbial succession will be critical for developing sustainable residue management strategies that promote long-term soil health and agroecosystem sustainability.

## Data Availability

The datasets presented in this study can be found in online repositories. The names of the repository/repositories and accession number(s) can be found at: https://www.ncbi.nlm.nih.gov/bioproject?LinkName=sra_bioproject&from_uid=30984231, PRJNA1053916.
