# Supplementary material for: Microbial community dynamics in rotational cropping: seasonality vs. crop-specific effects
Source: Front Microbiol. 2025 Oct 13;16:1675394. doi: 10.3389/fmicb.2025.1675394 (PMC12554655; doi:10.3389/fmicb.2025.1675394)
Supplement: Supplementary file 1 [file Data_Sheet_1.docx]

Supplementary Material

## Supplementary Figures


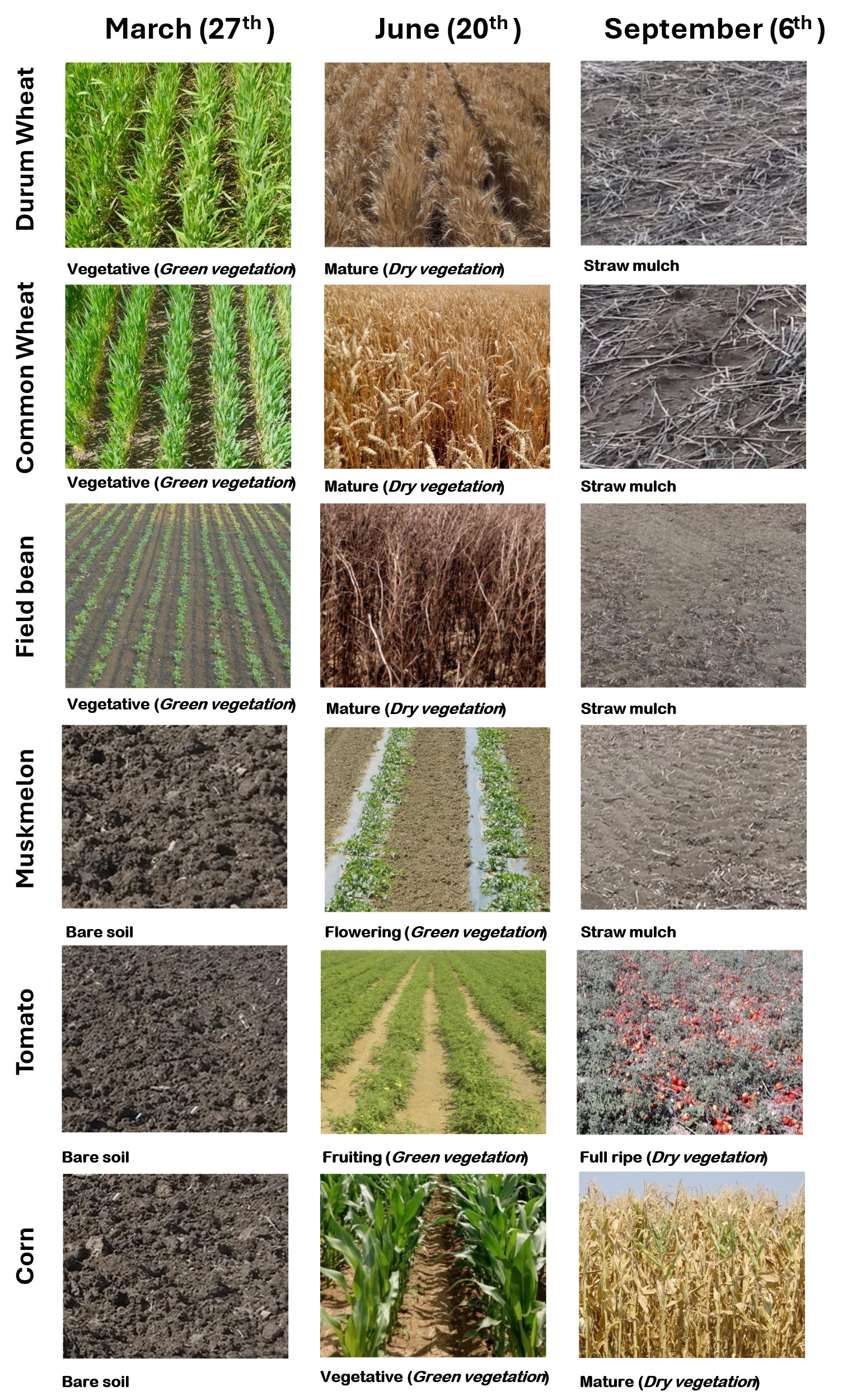


**Supplementary figure 1:** Phenological development of six crop species observed at three soil sampling dates during the 2019 growing season. Each row corresponds to a different crop species (Durum wheat, Common wheat, Field bean, Tomato, Corn, and Muskmelon), and each column to a distinct sampling date (March, June, and September), illustrating key growth stages from early vegetative development to maturity and post-harvest.

**
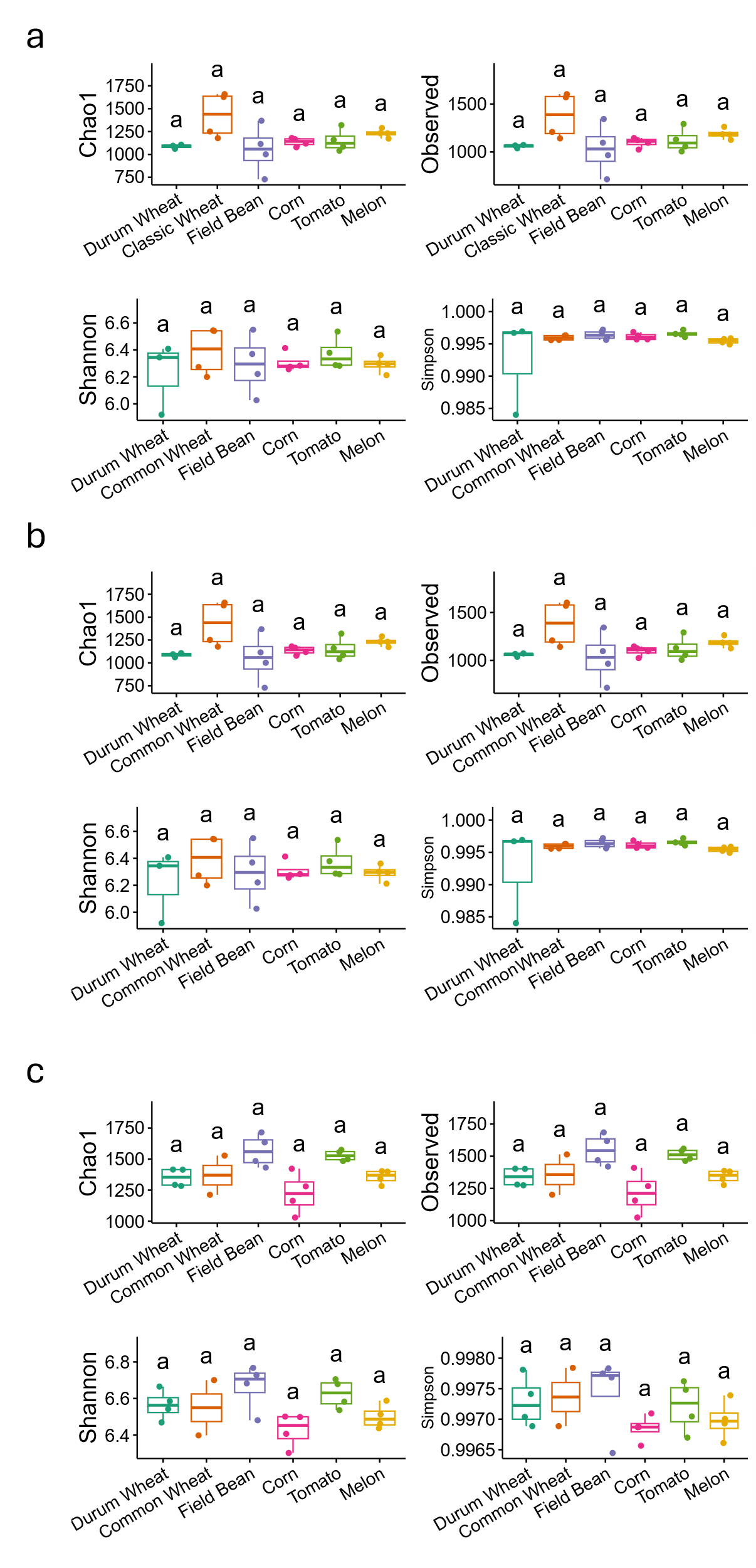
**

**Supplementary figure 2:** alpha diversity of all crop species divided by sampling time, a) March, b) June and c) September. The alpha diversity indices calculated are Shannon, Chao1, Obeserved and Simpson. Statistical significance was assessed with anova.


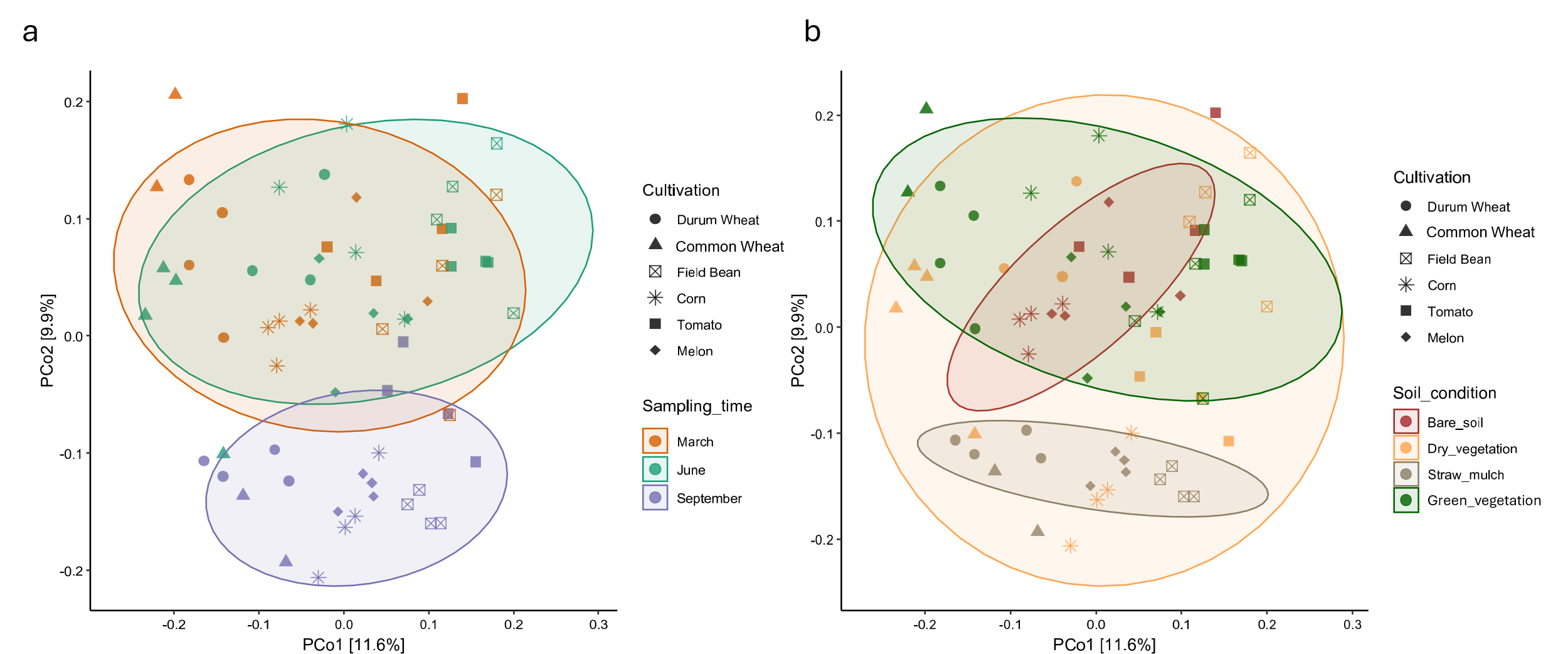


**Supplementary figure 3:** Principal Coordinates Analysis (PCoA) based on Bray-Curtis dissimilarity of microbial community composition across different sampling times (panel a) and soil conditions (panel b). Clustering patterns based on multivariate data analysis highlight distinct groupings among samples, differentiated by shapes and colors. Ellipses indicate 95% confidence intervals for each group, capturing the spread and central tendency of samples.


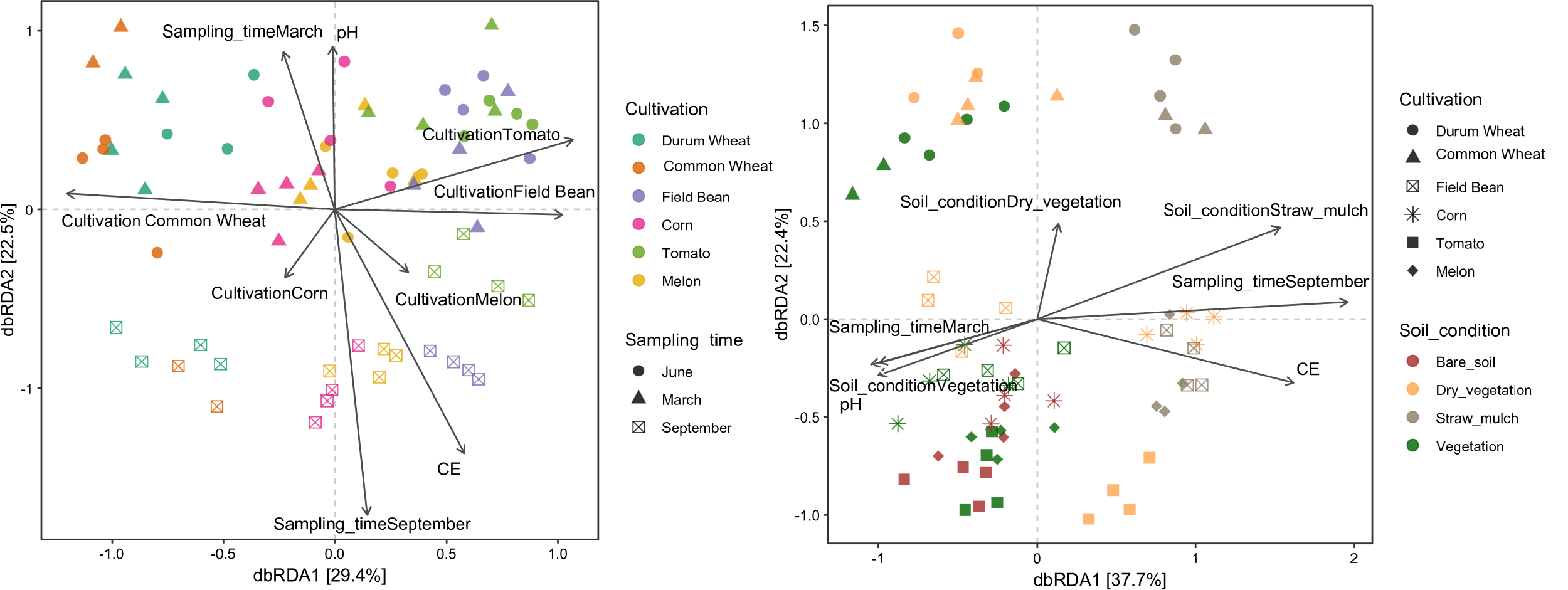


**Supplementary figure 4:** Principal Component Analysis (PCA) biplots illustrating multivariate relationships among samples based on cultivation type, sampling time, and soil condition. (a) Samples are differentiated by cultivation type (Durum Wheat, Common Wheat, Field Bean, Corn, Tomato, Melon) and sampling time (June, March, September), with shapes indicating sampling periods and colors denoting cultivation. (b) Samples are stratified by soil condition (Bare soil, Dry vegetation, Straw mulch, Vegetation) and cultivation type, with colors representing soil treatments and shapes indicating crops. Arrows represent principal component loadings, reflecting the magnitude and direction of variable influence

Supplementary table 1: pH and CE mean values for all samples.

|  | **Sampling_time** | **Cultivation** | **pH** | **CE** |
| --- | --- | --- | --- | --- |
| 1 | March | Durum Wheat | 8.12 | 0.1186 |
| 2 | March | Durum Wheat | 7.47 | 0.1182 |
| 3 | March | Durum Wheat | 7.97 | 0.1177 |
| 4 | March | Durum Wheat | 8.12 | 0.1245 |
| 5 | March | Classic Wheat | 8.14 | 0.1241 |
| 6 | March | Classic Wheat | 8.17 | 0.1177 |
| 7 | March | Classic Wheat | 8.18 | 0.1134 |
| 8 | March | Classic Wheat | 8.18 | 0.1162 |
| 9 | March | Melon | 8.28 | 0.1307 |
| 10 | March | Melon | 8.16 | 0.1274 |
| 11 | March | Melon | 7.77 | 0.1347 |
| 12 | March | Melon | 8.22 | 0.1217 |
| 13 | March | Tomato | 8.01 | 0.1312 |
| 14 | March | Tomato | 8.22 | 0.1214 |
| 15 | March | Tomato | 8.14 | 0.1229 |
| 16 | March | Tomato | 8.04 | 0.1174 |
| 17 | March | Corn | 8.09 | 0.1295 |
| 18 | March | Corn | 8.20 | 0.1226 |
| 19 | March | Corn | 8.2 | 0.1240 |
| 20 | March | Corn | 8.04 | 0.1241 |
| 21 | March | Field Bean | 7.51 | 0.1232 |
| 22 | March | Field Bean | 8.15 | 0.1161 |
| 23 | March | Field Bean | 8.08 | 0.1203 |
| 24 | March | Field Bean | 8.11 | 0.1195 |
| 25 | June | Durum Wheat | 7.89 | 0.1104 |
| 26 | June | Durum Wheat | 8.00 | 0.1201 |
| 27 | June | Durum Wheat | 7.89 | 0.1157 |
| 28 | June | Durum Wheat | 7.93 | 0.1154 |
| 29 | June | Classic Wheat | 7.92 | 0.1246 |
| 30 | June | Classic Wheat | 7.95 | 0.1262 |
| 31 | June | Classic Wheat | 7.92 | 0.1196 |
| 32 | June | Classic Wheat | 7.97 | 0.1250 |
| 33 | June | Melon | 8.56 | 0.1369 |
| 34 | June | Melon | 8.22 | 0.1301 |
| 35 | June | Melon | 8.03 | 0.1279 |
| 36 | June | Melon | 8.08 | 0.1257 |
| 37 | June | Tomato | 7.97 | 0.1461 |
| 38 | June | Tomato | 7.59 | 0.1800 |
| 39 | June | Tomato | 8.12 | 0.1284 |
| 40 | June | Tomato | 8.06 | 0.1265 |
| 41 | June | Corn | 8.03 | 0.1409 |
| 42 | June | Corn | 8.08 | 0.1259 |
| 43 | June | Corn | 8.01 | 0.1302 |
| 44 | June | Corn | 8.13 | 0.1269 |
| 45 | June | Field Bean | 8.06 | 0.1272 |
| 46 | June | Field Bean | 7.85 | 0.1538 |
| 47 | June | Field Bean | 8.01 | 0.1232 |
| 48 | June | Field Bean | 8.32 | 0.1230 |
| 49 | September | Durum Wheat | 7.78 | 0.1687 |
| 50 | September | Durum Wheat | 7.82 | 0.1642 |
| 51 | September | Durum Wheat | 7.78 | 0.1700 |
| 52 | September | Durum Wheat | 7.99 | 0.1613 |
| 53 | September | Classic Wheat | 7.63 | 0.1556 |
| 54 | September | Classic Wheat | 7.81 | 0.1595 |
| 55 | September | Classic Wheat | 7.72 | 0.1576 |
| 56 | September | Classic Wheat | 7.72 | 0.1576 |
| 57 | September | Melon | 7.09 | 0.1772 |
| 58 | September | Melon | 7.67 | 0.1927 |
| 59 | September | Melon | 7.89 | 0.1622 |
| 60 | September | Melon | 7.84 | 0.1609 |
| 61 | September | Tomato | 8.07 | 0.1587 |
| 62 | September | Tomato | 7.98 | 0.2195 |
| 63 | September | Tomato | 7.35 | 0.1720 |
| 64 | September | Tomato | 7.96 | 0.1780 |
| 65 | September | Corn | 8.07 | 0.1406 |
| 66 | September | Corn | 7.75 | 0.2100 |
| 67 | September | Corn | 7.75 | 0.1507 |
| 68 | September | Corn | 7.54 | 0.1458 |
| 69 | September | Field Bean | 7.04 | 0.2260 |
| 70 | September | Field Bean | 7.92 | 0.2280 |
| 71 | September | Field Bean | 7.93 | 0.1646 |
| 72 | September | Field Bean | 7.86 | 0.2115 |
